# *Toxoplasma gondii* induces FAK-Src-STAT3 signaling during infection of host cells that prevents parasite targeting by autophagy

**DOI:** 10.1371/journal.ppat.1006671

**Published:** 2017-10-16

**Authors:** Jose-Andres C. Portillo, Luis Muniz-Feliciano, Yalitza Lopez Corcino, So Jung Lee, Jennifer Van Grol, Sarah J. Parsons, William P. Schiemman, Carlos S. Subauste

**Affiliations:** 1 Department of Medicine, Division of Infectious Disease and HIV Medicine, Case Western Reserve University School of Medicine, Cleveland, OH, United States of America; 2 Department of Pathology, Case Western Reserve University School of Medicine, Cleveland, OH, United States of America; 3 Department of Microbiology and Cancer Center, University of Virginia, Charlottesville, VA, United States of America; 4 Case Comprehensive Cancer Center, Case Western Reserve University, Cleveland, OH, United States of America; University of Medicine and Dentistry of New Jersey, UNITED STATES

## Abstract

Targeting of *Toxoplasma gondii* by autophagy is an effective mechanism by which host cells kill the protozoan. Thus, the parasite must avoid autophagic targeting to survive. Here we show that the mammalian cytoplasmic molecule Focal Adhesion Kinase (FAK) becomes activated during invasion of host cells. Activated FAK appears to accompany the formation of the moving junction (as assessed by expression the parasite protein RON4). FAK activation was inhibited by approaches that impaired β1 and β3 integrin signaling. FAK caused activation of Src that in turn mediated Epidermal Growth Factor Receptor (EGFR) phosphorylation at the unique Y845 residue. Expression of Src-resistant Y845F EGFR mutant markedly inhibited ROP16-independent activation of STAT3 in host cells. Activation of FAK, Y845 EGFR or STAT3 prevented activation of PKR and eIF2α, key stimulators of autophagy. Genetic or pharmacologic inhibition of FAK, Src, EGFR phosphorylation at Y845, or STAT3 caused accumulation of the autophagy protein LC3 and LAMP-1 around the parasite and parasite killing dependent on autophagy proteins (ULK1 and Beclin 1) and lysosomal enzymes. Parasite killing was inhibited by expression of dominant negative PKR. Thus, *T*. *gondii* activates a FAK→Src→Y845-EGFR→STAT3 signaling axis within mammalian cells, thereby enabling the parasite to survive by avoiding autophagic targeting through a mechanism likely dependent on preventing activation of PKR and eIF2α.

## Author summary

*Toxoplasma gondii* is a protozoan that resides within host cells. Avoiding lysosomal degradation including that mediated by autophagy is central to the ability of *T*. *gondii* to survive within these cells. We uncovered that during the process of active invasion of host cells, *T*. *gondii* activates in a broad range of mammalian cells a signaling cascade downstream of FAK-Src that prevents targeting of the intracellular parasite by autophagy enabling its survival. This pathway is different from the previously identified survival strategy dependent upon parasite micronemal proteins-mediated EGFR autophosphorylation and Akt activation. Importantly, preventing *T*. *gondii*-induced manipulation of host cell signaling led to parasite killing in host cells that were not activated by immune modulators. Pharmacologic approaches that impair parasite-induced activation of host cell signaling that counter-regulates autophagy may lead to novel treatment against toxoplasmosis.

## Introduction

*Toxoplasma gondii* is an obligate intracellular protozoan that can cause disease in humans, including retinochoroiditis and encephalitis. *T*. *gondii* actively invades host cells, a process powered by the parasite’s own motility that is dependent on the sequential secretion of proteins present in the apical organelles called micronemes and rhoptries [1–3]. Micronemal proteins (MIC) act as adhesins that interact with the host cell membrane and also function through their association with the parasite glideosome that powers motility [2]. A complex of rhoptry neck proteins (RON) are secreted into the host cell membrane anchoring the parasite to the cell being invaded [1–3]. This complex contains RON2 that associates with the host cell membrane, plus RON4, RON5 and RON8 that are exposed to the host cell cytoplasm [1–3]. The complex forms a structure called moving or tight junction through which the parasite penetrates the host cell causing invagination of the host cell membrane [1–3]. Once invasion is completed, *T*. *gondii* dissociates from the host cell membrane and resides within a specialized niche called parasitophorous vacuole (PV).

*T*. *gondii* cannot withstand the lysosomal environment. The PV enables parasite survival since it is devoid of proteins required for fusion with endosomes and lysosomes [4]. However, in addition to the classical endosomal-lysosomal pathway, macroautophagy (commonly referred as autophagy) is an important constitutive mechanism that targets organelles and portions of cytoplasm to lysosomal degradation [5]. This indicates that *T*. *gondii* must avoid autophagic targeting as a survival mechanism within host cells.

Autophagy is characterized by the recruitment of Atg proteins to the isolation membrane that encircles a portion of the cell leading to the formation of an autophagosome [5]. The process is driven by the activation of the Unc-51-like kinase 1/2 (ULK1/2) complex and Beclin 1 –Phosphatidylinositol 3-kinase catalytic subunit type 3 (PI3KC3) complex, and is inhibited by activation of the mechanistic target of rapamycin complex 1 (mTORC1) [6–8]. We previously demonstrated that *T*. *gondii* induces autophosphorylation of epidermal growth factor receptor (EGFR) in host cells, a process mediated by MIC3 and MIC6, parasite proteins with EGF-like domains [9]. EGFR autophosphorylation is followed by activation of Akt (a molecule known to inhibit autophagy by activating mTORC1 [10]) and inhibition of targeting of the PV by autophagosomes [9]. However, autophagy is regulated at various levels by an array of signaling molecules. The efficiency by which *T*. *gondii* avoids autophagic targeting raised the possibility that the parasite acts at more than one level to successfully impair autophagic killing. Herein, we report that during the process of invasion by *T*. *gondii*, the mammalian cytoplasmic molecule Focal Adhesion Kinase (FAK) became activated at a site that appears to associate with RON4 (host cell-parasite junction). FAK activation led to activation of Src and its transactivation of EGFR that, instead of inducing Akt activation, triggered Y705 phosphorylation and activation of Signal Transducer and Activator of Transcription 3 (STAT3). In turn, STAT3 signaling prevented autophagic killing of the parasite likely by averting activation of Protein Kinase Double-stranded RNA-dependent (PKR) and its downstream effector Eukaryotic Initiation Factor 2α (eIF2α). Blockade of the FAK→Src→Y845-EGFR→STAT3 signaling cascade led to parasite killing in host cells that were not activated by immune modulators.

## Results

### *T*. *gondii* induces Src activation in mammalian cells

**Fig 1:**
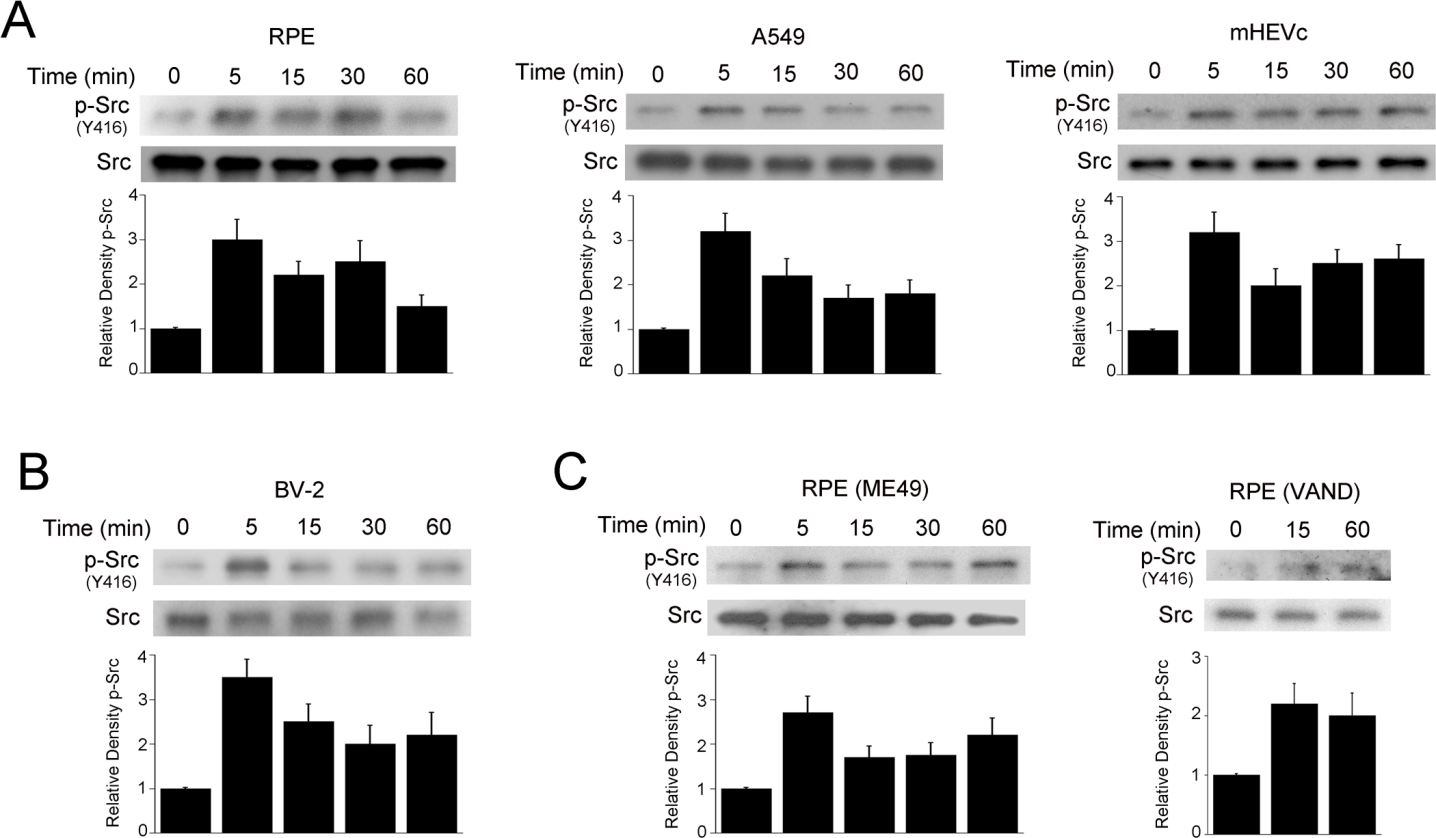
*T*. *gondii* induces Src activation in mammalian cells. *A*, Human retinal pigment epithelial cells (RPE), human lung epithelial cells (A549) and mouse endothelial cells (mHEVc) were challenged with tachyzoites of the RH (type I) strain of *T*. *gondii*. Cell lysates were obtained at the indicated time points and used to probe for total Src and phospho-Y416 Src. *B*, Mouse microglia (BV-2) were challenged with RH *T*. *gondii* tachyzoites as above. C, RPE were incubated with tachyzoites of the M49 or VAND strains of *T*. *gondii* followed by assessment of Src phosphorylation. Densitometry data represent means ± SEM of 3–4 independent experiments. Relative density of phospho-Src signal was obtained by normalization to total Src signal followed by normalization relative to the uninfected control samples (0 min time-point). Relative density of phospho-Src for uninfected samples was given a value of 1.

Src constitutes a signaling node that regulates multiple cellular processes [11]. Src activity is regulated by phosphorylation of tyrosine residues. Phosphorylation at Y416 in the activation loop in the kinase domain locks the catalytic domain into a fully active conformation resulting in high kinase activity [12]. Incubation of human retinal pigment epithelial cells (RPE) with tachyzoites of the RH strain of *T*. *gondii* (type I strain) resulted in an increased phosphorylation of Src at Y416 (Fig 1A). Similar results were observed in human lung epithelial cells (A549) and mouse endothelial cells (mHEVc) (Fig 1A). *T*. *gondii* enhanced Y416 Src phosphorylation not only in non-hematopoietic cells but also in a mouse microglia cell line (BV-2) (Fig 1B). In addition, increased Y416 phosphorylation was detected in cells infected with tachyzoites of the ME49 (type II strain) or VAND (atypical) strain (Fig 1C). Subsequent experiments were conducted using a type I strain (RH) unless otherwise stated. Taken together, different strains of *T*. *gondii* induce rapid activation of Src in various hematopoietic and non-hematopoietic cells.

### Formation of the moving junction during invasion of host cells appears to associate with FAK activation that triggers Src signaling

*T*. *gondii* activates EGFR in mammalian cells [9] and EGFR signaling can activate Src [13]. However, *T*. *gondii* infection of CHO cells (EGFR null) still triggered Y416 Src phosphorylation, indicating that parasite-induced Src activation can occur independently of EGFR (S1A Fig). *T*. *gondii* is reported to induce G protein-coupled receptor (GPCR), TLR2 and TLR4 signaling [14, 15], molecules that signal through Src [16–18]. However, while treatment of A549 cells with pertussis toxin (PTx, an inhibitor of GPCR signaling) markedly impairs Akt phosphorylation induced by Lysophosphatidic acid (LPA, a ligand for GCPR), PTx did not inhibit parasite-induced phosphorylation of Src Y416 (S1B Fig). Similarly, knockdown of MyD88 (adaptor protein that mediates TLR2/4 signaling) in A549 cells did not inhibit parasite-induced phosphorylation of Src Y416 or affect the ability of tachyzoites to infect mammalian cells (S1C Fig).

The parasite kinases, rhoptry protein 16 (ROP16) and ROP18, are delivered into the host cells and induce tyrosine phosphorylation of STAT3/6 and threonine phosphorylation of Immunity Related GTPases (IRG), respectively in infected mammalian cells [19–22]. To examine the potential role of these proteins in Src activation, A549 cells were infected with tachyzoites deficient in these proteins (*Δ*r*op16* or *Δ*r*op18*) or their WT controls. Infection with *Δ*r*op16* or *Δ*r*op18* tachyzoites did not diminish Src Y416 phosphorylation (S1D Fig). Altogether, *T*. *gondii* can trigger phosphorylation of Src Y416 independently of EGFR, GPCR and TLR signaling as well as independently of ROP16 and ROP18.

**Fig 2:**
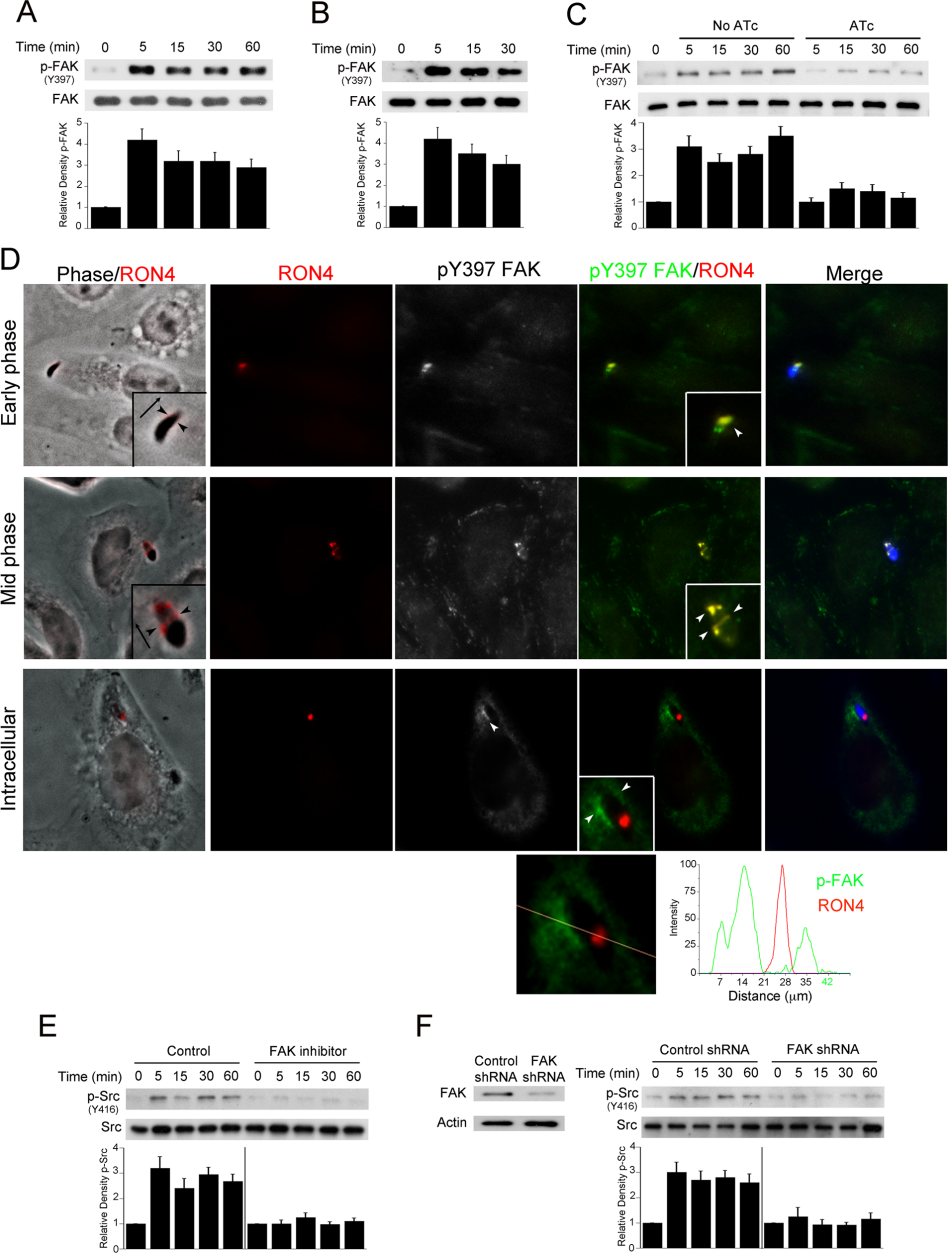
FAK mediates Src activation during *T*. *gondii* infection. *A*, *B*, RPE cells (*A*) or CHO cells (*B*) were challenged with RH *T*. *gondii* for the indicated time points. Cell lysates were used to examine expression of total FAK and phospho-Y397 FAK by immunoblot. *C*, MIC8KOi parasites were cultured in HFF with or without anhydrotetracycline (ATc; 1 μg/ml). Tachyzoites were harvested and incubated with A549 cells for the indicated time points. Expression of total FAK and phospho-Y397 FAK were assessed by immunoblot. Relative densities of phospho-FAK were compared to that of uninfected (control) cells. Relative density of phospho-FAK for uninfected samples was given a value of 1 Densitometry data represent means ± SEM of 3 independent experiments. *D*, CHO cells were incubated with YFP *T*. *gondii* (RH) for 2.5 min followed by assessment of phospho-Y397 FAK and RON4 expression by immunofluorescence. *T*. *gondii* was pseudocolored blue. Arrowheads indicate co-localization of RON4 with phospho-Y397FAK or accumulation of phospho-Y397 FAK around an intracellular tachyzoite. Original magnification X630. Quantification of phospho-Y397 FAK and RON4 signaling intensity for the intracellular parasite is shown underneath. *E*, A549 cells were incubated with vehicle or FAK inhibitor (1 μM) prior to challenge with RH *T*. *gondii*. Cell lysates were obtained and used to probe for total Src and phospho-Y416 Src. A vertical line was inserted between densitometry data of lysates from control and FAK inhibitor-treated cells to indicate that relative densities of phospho-FAK from infected cells treated with or without FAK inhibitor were compared to bands from their respective uninfected (control) cells. Relative density of phospho-FAK for uninfected samples was given a value of 1. *F*, mHEVc cells transduced with lentiviral vectors that express either FAK shRNA or control shRNA were incubated with RH *T*. *gondii*. Immunoblots and densitometries were assessed as above. Experiments shown are representative of 3–4 independent experiments.

FAK is a widely expressed cytoplasmic non-receptor tyrosine kinase that activates Src [23]. Y397 is the major autophosphorylation site of FAK that correlates with increased catalytic activity and creates a high-affinity binding site for the SH2 domain of Src [24–26]. We examined whether *T*. *gondii* causes FAK phosphorylation at Y397 and whether FAK mediates parasite-induced Src activation. *T*. *gondii* induced rapid phosphorylation of Y397 in FAK in RPE, A549, mHEVc and CHO cells (Fig 2A–2F). The experiments with CHO cells indicate that, similar to Src, *T*. *gondii* can activate FAK in the absence of EGFR (Fig 2B). Next, we used tetracycline-induced conditional micronemal protein 8 knockout parasites (MIC8KOi) to determine whether host cell invasion is required for phosphorylation of Y397. Incubation with aminotetracycline (ATc) led to the phenotype of MIC8 deficiency—parasites with markedly impaired ability to infect host cells [27] (A549 cells; no ATC: 28.3 ± 4.3% and 30.3 ± 5% of infected cells at 2 and 18 hr respectively; ATc: 3.3 ± 0.5% and 3.6 ± 0.6% at 2 and 18 hr respectively). In contrast to MIC8-sufficient parasites, tachyzoites deficient in MIC8 failed to induce phosphorylation of FAK Y397 (Fig 2C). FAK bridges extracellular stimuli with Src signaling. Thus, we examined if phosphorylation of FAK occurred at the site of parasite-host cell interaction. CHO cells were challenged with *T*. *gondii-*YFP followed by staining with Abs against anti-phospho-Y397 FAK and RON4, a central component of the moving junction. This structure manifests initially as a cap around the apical tip of the tachyzoite and subsequently, as a ring around the invading parasite. During early stages of parasite invasion, enhanced activation of FAK (*i*.*e*., increased Y397 phosphorylation) appeared to associate with RON4 around the apical pole of parasites although the overlap may not be complete (Fig 2D, early phase). Increased phospho-Y397 FAK expression also co-localized with ring structures that expressed RON4 (Fig 2D, mid phase). Finally, increased expression of phospho-Y397 FAK was also noted around parasites that had completed invasion (Fig 2D, intracellular). Altogether, our findings indicate that *T*. *gondii* induces FAK activation during the process of invasion of host cells.

A549 cells were treated with vehicle or FAK inhibitor (PF-573228), followed by challenge with *T*. *gondii* to test whether FAK mediates *T*. *gondii*-induced Src activation. The FAK inhibitor markedly impaired parasite-induced phosphorylation of Src Y416 (Fig 2E). This occurred despite the fact that the FAK inhibitor did not impair the ability of tachyzoites to infect cells. Similarly, knockdown of FAK markedly reduced Y416 phosphorylation of Src in mHEVc cells challenged with *T*. *gondii* (Fig 2F). Thus, infection with *T*. *gondii* causes Y397 phosphorylation of FAK that appears to associate with RON4, a component of the moving junction. In turn, FAK mediates parasite-induced Src activation.

### Inhibition of β integrin signaling prevents *T*. *gondii*-induced FAK activation

**Fig 3:**
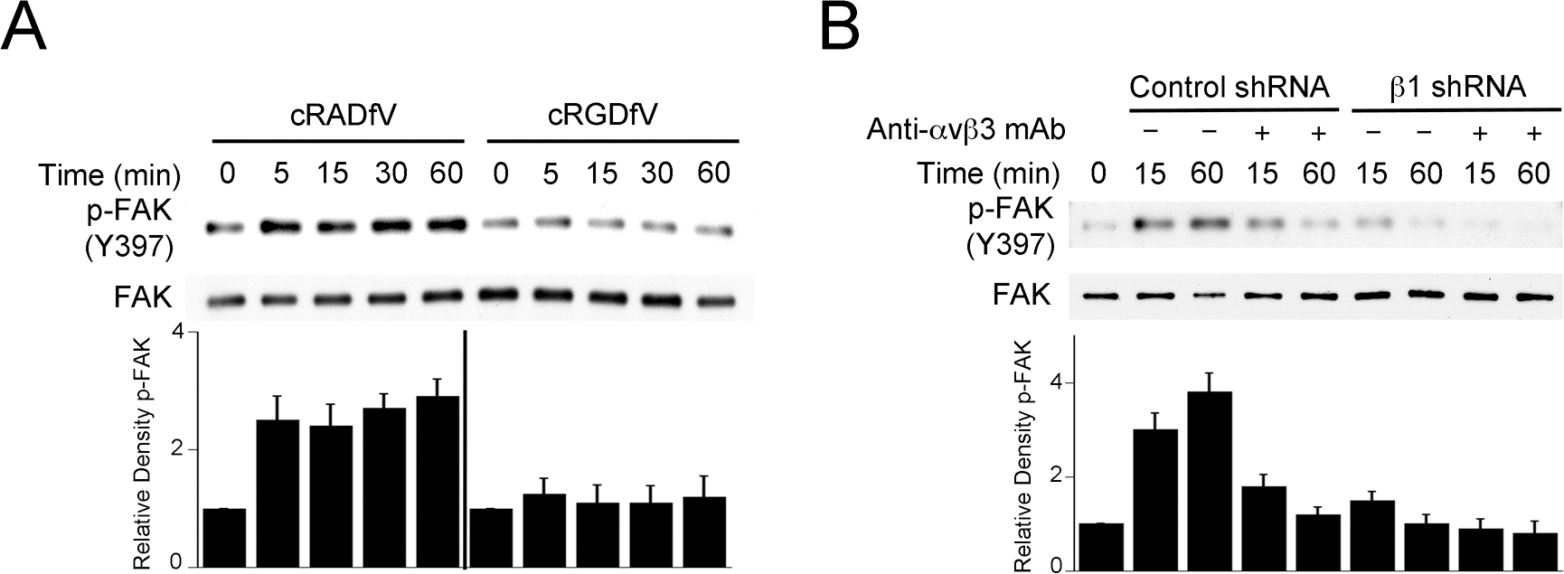
Inhibition of β integrin signaling impairs *T*. *gondii*-induced FAK activation. *A*, RPE were incubated with cRADfV or cRGDfV prior to challenge with RH *T*. *gondii*. Total FAK and phospho-Y397 were assessed by immunoblot. A vertical line was inserted between densitometry data of lysates from cRADfV- and cRGDfV-treated cells to indicate that relative densities of phospho-FAK from infected cells were compared to bands from their respective uninfected (control) cells. Relative density of phospho-FAK for uninfected samples was given a value of 1. *B*, MDA-MB-231 human breast epithelial cells transduced with vector encoding control shRNA or β1 integrin shRNA were incubated with or without a neutralizing anti-α_v_β3 mAb prior to challenge with RH *T*. *gondii*. Total FAK and phospho-Y397 were assessed by immunoblot. Densitometries were calculated as above. Densitometry data represent means ± SEM of 3 independent experiments.

Integrin clustering causes recruitment of FAK to the intracytoplasmic tail of integrins leading to FAK autophosphorylation at Y397 and FAK activation [25, 28]. We took 3 approaches to examine the role of β integrins in *T*. *gondii*-induced FAK activation. First, we incubated mammalian cells with cRGDfV, a peptide that binds α_v_β integrins inhibiting their interaction to ligands [29]. cRADfV peptide was used as control. As shown in Fig 3A, cRGDfV peptide markedly diminished Y397 phosphorylation of FAK in RPE cells challenged with *T*. *gondii*. Next, we examined the effects of knockdown of β1 integrin or of treatment with a neutralizing anti-α_v_β3 Ab. We used MDA-MB-231 cells transduced with lentiviral vector encoding shRNA against β1 integrin (proven to be deficient in β1 integrin expression [30]) and MB-MB-231 cells transduced with a lentiviral vector encoding control shRNA. MDA-MB-231 cells deficient in β1 integrin exhibited reduced FAK Y397 phosphorylation after *T*. *gondii* infection (Fig 3B). Incubation with a neutralizing anti-α_v_β3 Ab decreased parasite-induced FAK Y397 phosphorylation both in cells sufficient and deficient in β1 integrin (Fig 3B). None of the approaches to inhibit β integrin signaling impaired the initial percentage of infected cells (see S2C and S2D Fig). Thus, FAK activation triggered by *T*. *gondii* infection appears to be dependent on β1 and β3 integrins.

### *T*. *gondii*-induced β integrin-FAK-Src signaling prevents autophagic killing of *T*. *gondii*

**Fig 4:**
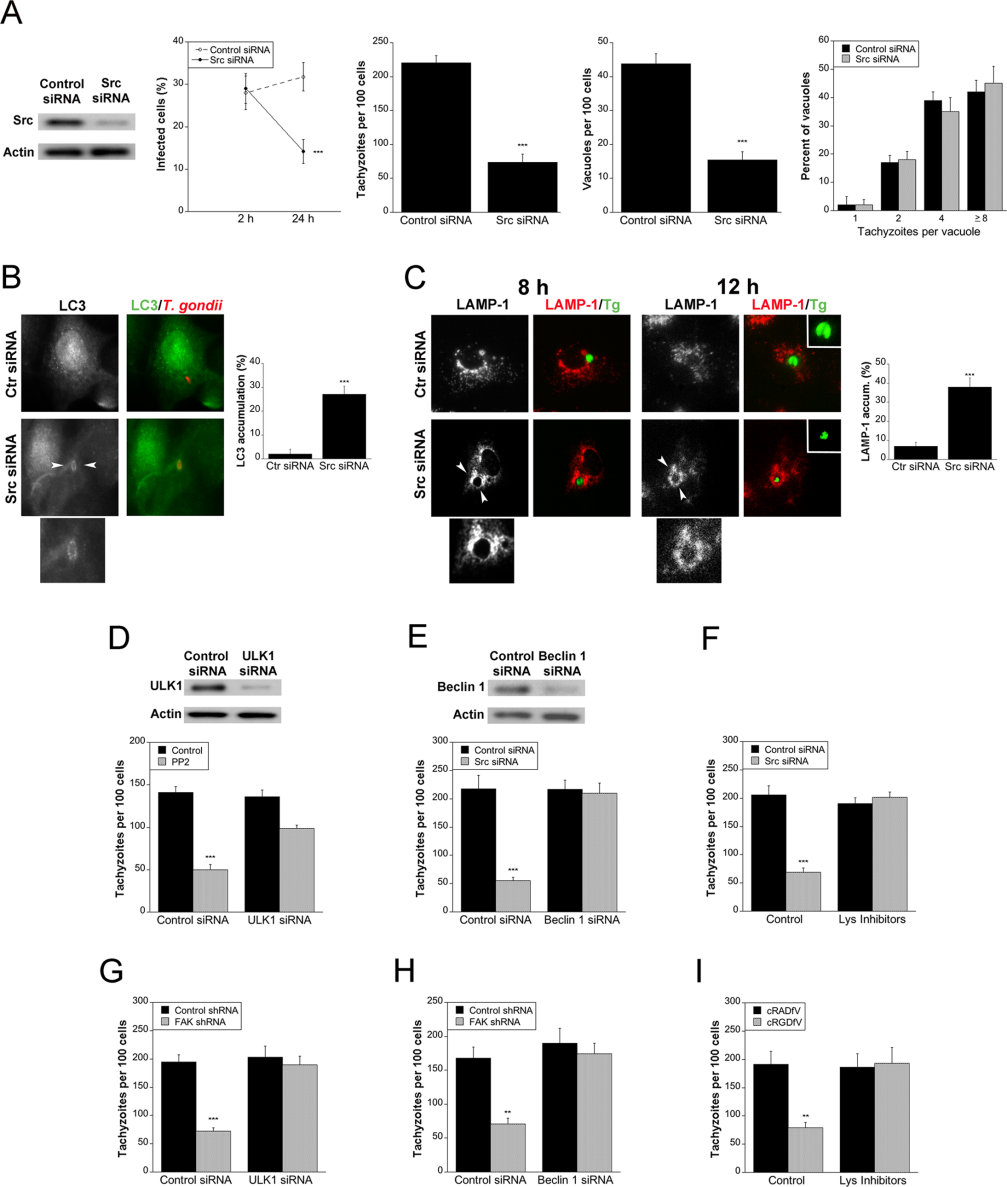
Blockade of Src induces accumulation of the autophagy protein LC3 around the parasite, vacuole-lysosome fusion and killing of the parasite dependent on autophagy the proteins ULK1 and Beclin 1. *A*, A549 cells were challenged with RH *T*. *gondii* after transfection with either control siRNA or Src siRNA. Monolayers were examined at 2 and 24 h to determine the percentages of infected cells, and at 24 h to ascertain the numbers of *T*. *gondii* tachyzoites, *T*. *gondii*-containing vacuoles per 100 cells and parasites per vacuole. *B*, A549 cells transfected with either control siRNA or Src siRNA were challenged with *T*. *gondii*-RFP (RH). Expression of LC3 was examined by immunofluorescence 5 h post-challenge. Arrowheads indicate accumulation of LC3 around the parasite. Original magnification X630. *C*, A549 cells transfected with either control siRNA or Src siRNA were challenged with *T*. *gondii*-YFP (RH). Expression of LAMP-1 was examined by immunofluorescence 8 h and 12 h post-challenge. Arrowheads indicate accumulation of LAMP-1 around the parasite. *D*, mHEVc cells were transfected with control siRNA or ULK1 siRNA followed by treatment with or without PP2 prior to challenge with RH *T*. *gondii*. Monolayers were examined by light microscopy 24 h post-infection. *E*, A549 cells transfected with control siRNA or Src siRNA were transfected with Beclin 1 siRNA. Cells were challenged with RH *T*. *gondii* and monolayers were examined by light microscopy 24 h post-infection. *F*, A549 cells transfected control siRNA or Src siRNA were infected with RH *T*. *gondii*. Leupeptin plus pepstatin (Lys Inhibitors) were added post-infection and monolayers were examined microscopically 24 h post-challenge. *G*-*I*, mHEVc cells transduced with lentiviral vectors that express either FAK shRNA or control shRNA were transfected with ULK1 siRNA (*G*), Beclin 1 siRNA (*H*) or control siRNA followed by incubation with RH *T*. *gondii*. mHEVc cells challenged with RH *T*. *gondii* were also incubated with leupeptin plus pepstatin (Lys Inhibitors) (*I*). Monolayers were examined microscopically 24 h post-challenge. Results are shown as the mean ± SEM of 3 independent experiments. ** *P* < 0.01; *** *P* < 0.001.

We examined whether *T*. *gondii* activates Src and FAK signaling in order to survive within mammalian cells. RPE, mHEVc and BV-2 cells were incubated with vehicle or PP2, a Src kinase inhibitor, followed by challenge with *T*. *gondii*. While PP2 did not impair the initial percentage of infected cells, PP2 significantly reduced the percentage of infected cells at 24 h, the number of tachyzoites per 100 cells and the numbers of *T*. *gondii*-containing vacuoles per 100 cells at 24 h (S2A Fig). Similar results were observed after knockdown of Src (Fig 4A). The *T*. *gondii*-containing vacuoles that persisted after Src knockdown had similar numbers of parasites as those from control siRNA-expressing cells indicating that the effect of Src knockdown was to induce parasite killing rather than restrict parasite replication (Fig 4A). Knockdown of FAK in mHEVc cells not only induced killing of RH *T*. *gondii* but also of the VAND strain of the parasite (S2B Fig). Similarly, incubation of RPE with cRGDfV peptide, knockdown of β1 integrin in MDA-MB-231 cells or incubation of these cells with a neutralizing anti-α_v_β3 Ab decreased the percentages of infected cells at 24 h and the numbers of parasites per 100 host cells (S2C and S2D Fig). Taken together, these findings revealed that parasite-induced β integrin→FAK→Src signaling prevents killing of *T*. *gondii* within host cells.

We examined whether parasite killing after blockade of Src and its upstream inducers is dependent on autophagy. The distribution of LC3, a protein associated with autophagosome membrane, was examined in *T*. *gondii*-infected cells rendered deficient in Src. A549 cells transfected with either control siRNA or Src siRNA followed by challenge with *T*. *gondii* and immunofluorescence using an anti-LC3 Ab. Knockdown of Src resulted in a significant accumulation of LC3 (ring) around the parasite (Fig 4B). Moreover, Src knockdown caused accumulation of the late endosomal/lysosomal marker LAMP-1 around *T*. *gondii* (Fig 4C). At later times post-infection (12 h), tachyzoites encircled by LAMP-1 exhibited morphology that suggested they were undergoing degradation (Fig 4C). To investigate whether *T*. *gondii* killing during blockade of Src is dependent on the autophagy machinery, we first examined the effects of knockdown of ULK1 and Beclin 1. ULK1 and the Beclin 1-PI3KC3 complex promote autophagosome formation and maturation [5]. Moreover, ULK1 is key for the stimulation of canonical autophagy in mammalian cells [6–8]. Knockdown of ULK1 or Beclin 1 prevented induction of anti-*T*. *gondii* activity in mammalian cells incubated with PP2 or transfected with Src siRNA (Fig 4D and 4E). We examined the effects of the lysosomal protease inhibitors leupeptin and pepstatin since autophagosomes deliver their cargo to lysosomes for degradation. Treatment with lysosomal inhibitors also ablated anti-*T*. *gondii* activity in Src deficient cells (Fig 4F). Similarly, anti-*T*. *gondii* activity induced in cells transduced with FAK shRNA-encoding lentiviral vector was ablated by knockdown of ULK1 or Beclin 1 (Fig 4G and 4H). Finally, lysosomal inhibitors prevented anti-*T*. *gondii* activity induced by cRGDfV peptide (Fig 4I). Thus, engagement of the β integrin→FAK→Src pathway is critical to promote parasite survival since it prevented *T*. *gondii* killing dependent on the autophagy machinery and lysosomal protease activity.

### Src signaling induced by *T*. *gondii* triggers trans-activation of EGFR that is required to prevent parasite killing within host cells

**Fig 5:**
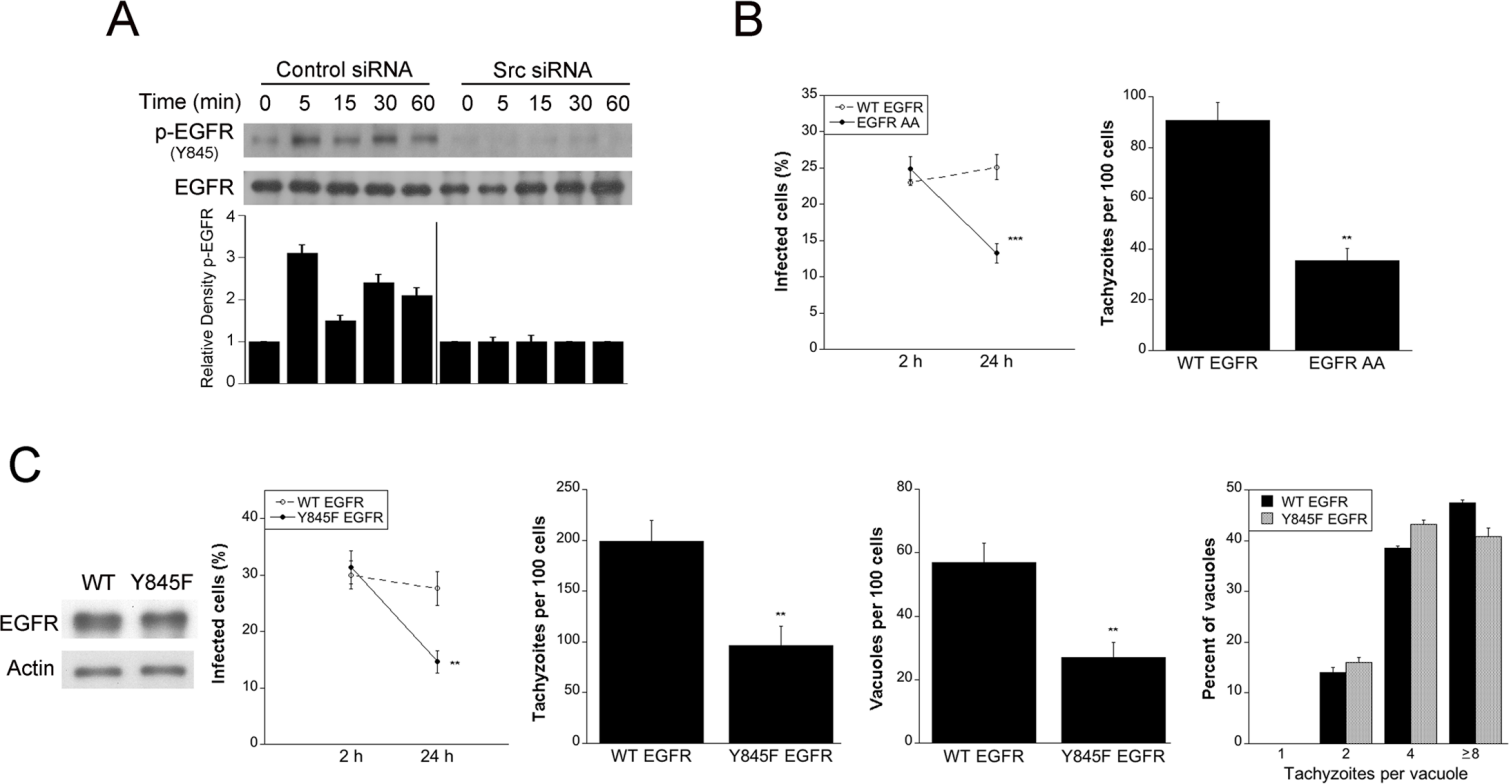
Src signaling induced by *T*. *gondii* triggers trans-activation of EGFR that is required to prevent parasite killing within host cells. *A*, A549 cells were transfected with control siRNA or Src siRNA followed by challenge with RH *T*. *gondii*. Expression of total EGFR and phospho-Y845 EGFR was assessed by immunoblot. A vertical line was inserted between densitometry data of lysates from cells transfected with control or Src siRNA to indicate that relative densities of phospho-Y845 EGFR from infected cells were compared to bands from their respective uninfected (control) cells. Relative density of phospho-Y845 EGFR for uninfected samples was given a value of 1. Densitometry data represent means ± SEM of 3 independent experiments. *B*, NMuMG with stable expression of WT EGFR or EGFR AA mutant were challenged with RH *T*. *gondii*. Monolayers were examined at 2 and 24 h to determine the percentages of infected cells and at 24 h to ascertain the numbers of *T*. *gondii* tachyzoites per 100 cells. *C*, A549 cells were transfected with plasmids encoding WT EGFR or Y845F EGFR followed by challenge with RH *T*. *gondii*. Monolayers were examined at 2 and 24 h to determine the percentages of infected cells, and at 24 h to ascertain the numbers of *T*. *gondii* tachyzoites, *T*. *gondii*-containing vacuoles per 100 cells and parasites per vacuole. Results are shown as the mean ± SEM of 3 independent experiments. ** *P* < 0.01; *** *P* < 0.001.

Src interacts with growth factor receptors including EGFR, a surface molecule engaged by *T*. *gondii* during host cell invasion [9]. The parasite adhesins MIC3 and MIC6 contain EGF-like domains and cause EGFR signaling that activates Akt and impairs autophagic targeting of the parasitophorous vacuole [9]. Src binds EGFR and transactivates this receptor by causing EGFR phosphorylation at a unique Y845 residue leading to signaling via alternate pathways downstream of EGFR [31–34]. We examined whether *T*. *gondii* induces EGFR phosphorylation at Y845. A549 cells expressing either control siRNA or Src siRNA were challenged with *T*. *gondii*. *T*. *gondii* induced EGFR Y845 phosphorylation, an effect that was ablated by knockdown of Src (Fig 5A). Next, we examined the relevance of parasite-induced Y845 EGFR phosphorylation. EGFR with dialanine substitution of L679 and L680 (EGFR AA) is defective in Src-induced Y845 phosphorylation [35]. NMuMG epithelial cells with stable expression of human WT EGFR or EGFR AA were infected with *T*. *gondii*. A significant reduction in the percentages of infected cells and the numbers of parasites per 100 cells at 24 h was detected in cells expressing EGFR AA (Fig 5B). To further explore the importance of Y845 EGFR phosphorylation, A549 cells were transfected with a plasmid encoding either WT EGFR or a phenylalanine substitution of Y845 (Y845F EGFR) [31, 32] followed by challenge with RH *T*. *gondii*. Y845F mutant EGFR does not signal through Y845 phosphorylation but retains full EGFR kinase activity [31, 32]. Cells transfected with Y845F-EGFR plasmid exhibited a significant decrease in the percentages of infected cells, the numbers of tachyzoites and vacuoles per 100 cells without affecting the number of tachyzoite per vacuole (Fig 5C). These findings indicate that preventing Y845 EGFR phosphorylation results in killing of *T*. *gondii* rather than reduction in intracellular replication of the parasite. Taken together, *T*. *gondii*-induced Src signaling causes EGFR transactivation that in turn prevents parasite killing.

### Src-mediated EGFR transactivation is not required for Akt activation triggered by *T*. *gondii* but mediates ROP16-independent STAT3 activation

**Fig 6:**
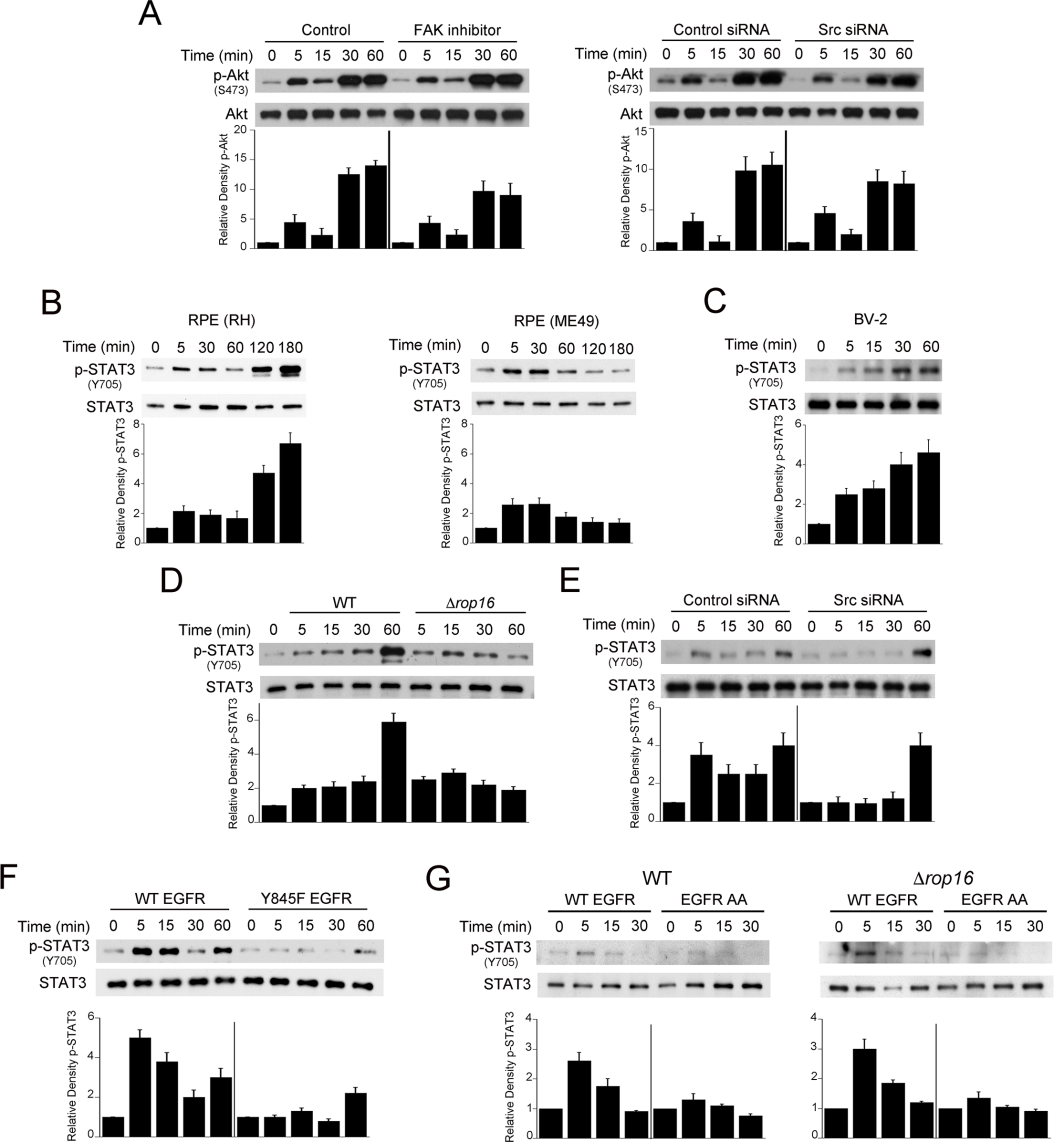
Src-mediated EGFR transactivation is not required for Akt activation triggered by *T*. *gondii* but mediates parasite-induced early STAT3 activation. *A*, A549 cells were treated with or without FAK inhibitor or were transfected with control siRNA or Src siRNA followed by challenge with RH *T*. *gondii*. Expression of total Akt and phospho-S473 Akt were examined by immunoblot. A vertical line was inserted between densitometry data of lysates from cells treated with control or FAK inhibitor, or transfected with control or Src siRNA to indicate that relative densities of phospho-Akt from infected cells were compared to bands from their respective uninfected (control) cells. Relative density of phospho-Akt for uninfected samples was given a value of 1. *B*, Human RPE cells were challenged with RH (type I) or ME49 (type II) strains of *T*. *gondii*. Expression of total STAT3 and phospho-Y705 STAT3 were examined by immunoblot. Relative densities of phospho-STAT3 in lysates from infected cells were compared to those from their respective uninfected (control) cells. Relative density of phospho-STAT3 for uninfected samples was given a value of 1. *C*, BV-2 cells were challenged with RH *T*. *gondii* followed by assessment of total STAT3 and phospho-Y705 STAT3 by immunoblot. *D*, A549 cells were challenged with either WT or *Δrop16 T*. *gondii*. Cell lysates were probed for total STAT3 and phospho-Y705 STAT3. Band densities in lysates from infected cells were compared to those from uninfected (control) cells. Densitometries for control bands were given a value of 1. *E*, A549 cells transfected with control siRNA or Src siRNA were challenged with RH *T*. *gondii*. Immunoblot and densitometries were assessed as above. *F*, A549 cells transfected with plasmid encoding either WT-EGFR or Y845F-EGFR were infected with *T*. *gondii* 48 h after transfection. Immunoblot and densitometries were assessed as above. *G*, NMuMG that express WT EGFR or EGFR AA mutant were challenged with WT or *Δrop16 T*. *gondii*. Immunoblot and densitometries were assessed as above. Densitometry data represent means ± SEM of 3 independent experiments.

Direct ligation of EGFR by MIC3 and MIC6 causes activation of Akt that prevents autophagic targeting of the parasite [9]. However, inhibition of FAK or Src knockdown did not significantly diminish the rapid S473 Akt phosphorylation induced by *T*. *gondii* infection of host cells (Fig 6A). Phosphorylation of the Y845 residue of EGFR activates signaling cascades including STAT3 [34]. Thus, we examined whether Src and EGFR Y845 mediate rapid STAT3 activation in *T*. *gondii*-infected cells. *T*. *gondii* is reported to induce Y705-STAT3 phosphorylation in macrophages, fibroblasts and 293T cells [20, 36–38]. Fig 6B–6G shows that tachyzoites of the RH or ME49 strains of *T*. *gondii* caused rapid Y705 STAT3 phosphorylation in RPE, A549, mHEVc, BV-2 and NMuMG cells.

The parasite kinase ROP16 is injected into the host cell cytoplasm, rapidly migrates to the nucleus and causes STAT3 activation. However, while ROP16 is reported to induce Y705 STAT3 phosphorylation as early 1.5 h post infection in macrophages, ROP16 did not appear to mediate STAT3 phosphorylation at earlier time points after infection in these cells [38]. To confirm these findings, we infected A549 epithelial cells with WT parasites or *Δrop16 T*. *gondii*. A549 cells infected with both types of parasites exhibited similar levels of Y705 STAT3 phosphorylation at the early time points post infection (Fig 6D). Lower STAT3 phosphorylation was noted at 1 h post-infection with Δ*rop16 T*. *gondii* (Fig 6D). Next, we examined the role of Src and EGFR transactivation in STAT3 activation induced by *T*. *gondii* infection. Epithelial cells expressing either Src siRNA or control siRNA were challenged with *T*. *gondii*. Knockdown of Src diminished the early Y705 STAT3 phosphorylation induced by the parasite (Fig 6E). In addition, the presence of EGFR in CHO cells markedly enhanced *T*. *gondii*-induced early Y705 STAT3 phosphorylation (S3A Fig). To examine whether EGFR Y845 phosphorylation promotes STAT3 activation triggered by *T*. *gondii*, human epithelial cells were transfected with a plasmid encoding WT EGFR or Y845F EGFR. Transfection with the Y845F EGFR plasmid markedly inhibited *T*. *gondii*-induced early Y705 STAT3 phosphorylation (Fig 6F). Finally, NMuMG epithelial cells that express EGFR AA (defective in Src-induced Y845 phosphorylation) exhibited reduced Y705 STAT3 phosphorylation after challenge with WT or *Δrop16 T*. *gondii* (Fig 6G). Taken together, *T*. *gondii-*induced Y845 EGFR signaling mediates ROP16-independent STAT3 activation.

### *T*. *gondii*-induced STAT3 signaling prevents autophagic killing of the parasite

**Fig 7:**
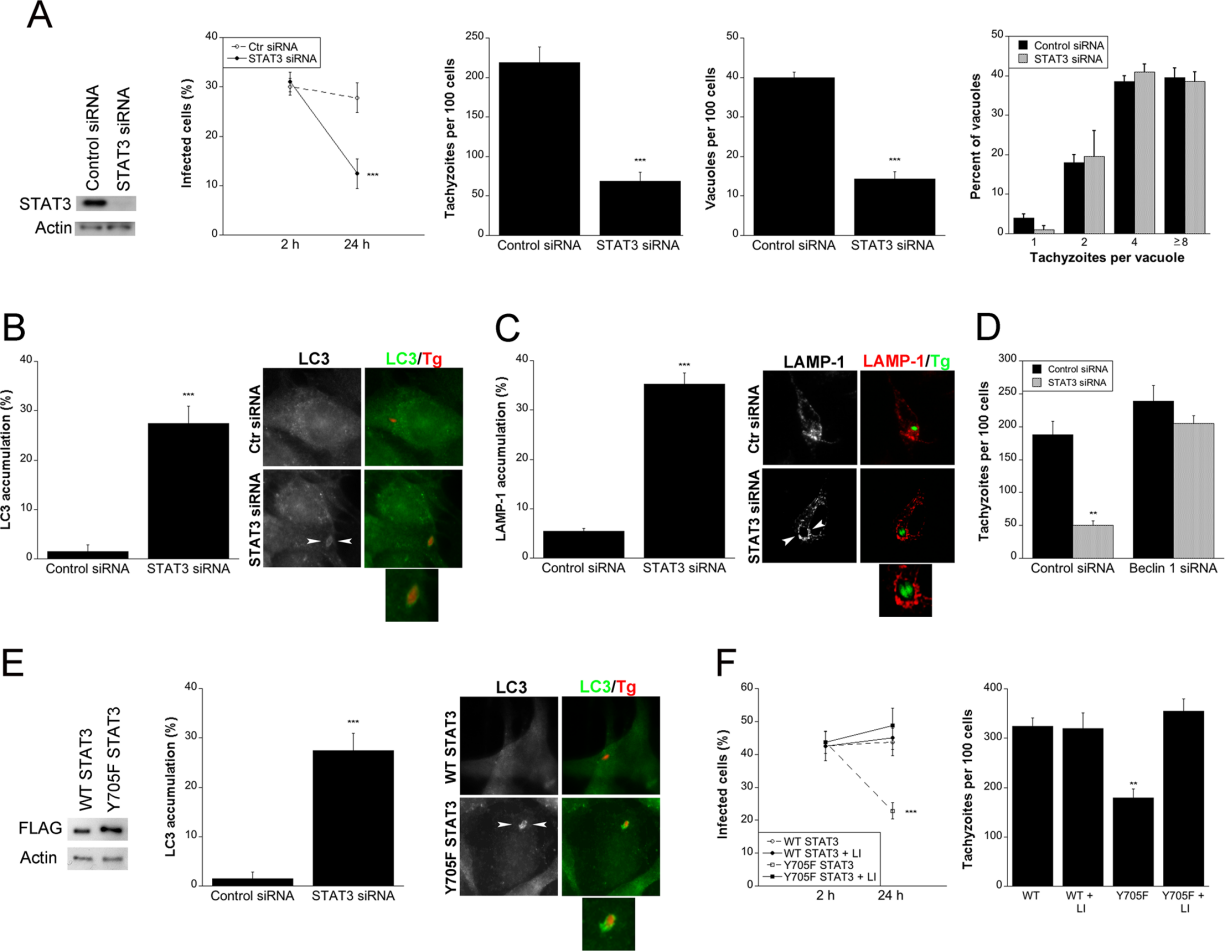
Src/EGFR-induced STAT3 signaling prevents autophagic killing of *T*. *gondii*. *A*, A549 cells were transfected with control siRNA or STAT3 siRNA and challenged with RH *T*. *gondii*. Monolayers were examined microscopically at 2 and 24 h to determine the percentages of infected cells, and at 24 h to ascertain the numbers of *T*. *gondii* tachyzoites, *T*. *gondii*-containing vacuoles per 100 cells and parasites per vacuole. *B*, A549 cells transfected with control siRNA or STAT3 siRNA were challenged with *T*. *gondii*-RFP (RH). Expression of LC3 was examined by immunofluorescence 5 h post-challenge to determine the percentage of cells with LC3 accumulation around the parasite. Arrowheads indicate accumulation of LC3 around the parasite. Original magnification X630. *C*, A549 cells transfected with control siRNA or STAT3 siRNA were challenged with *T*. *gondii*-YFP (RH). Expression of LAMP-1 was examined by fluorescence microscopy 8 h post-challenge. The percentage of cells with LAMP-1 accumulation around the parasite was determined. Arrowheads indicate accumulation of LAMP-1 around the parasite. *D*, A549 cells transfected with Beclin 1 siRNA were transfected with STAT3 siRNA followed by challenge with RH *T*. *gondii*. Monolayers were examined 24 h post-challenge. *E*, mHEVc cells transfected with WT STAT3 or Y705F STAT3 were challenged with *T*. *gondii*-RFP (RH). Expression of LC3 was examined by immunofluorescence 5 h post-challenge. LC3 accumulation around the parasite was assessed as above. *F*, mHEVc cells transfected with WT STAT3 or Y705F STAT3 were challenged with RH *T*. *gondii* followed by addition of leupeptin/pepstatin (lysosomal inhibitors, LI). Monolayers were examined at 2 and 24 h post-challenge. Results are shown as the mean ± SEM of 3 independent experiments. ** *P* < 0.01; *** *P* < 0.001.

STAT3 can inhibit autophagy [39, 40]. Thus, we examined whether blockade of STAT3 triggered autophagy-dependent killing of *T*. *gondii*. Human epithelial cells were transfected with STAT3 siRNA or control siRNA followed by incubation with *T*. *gondii* tachyzoites. Knockdown of STAT3 resulted in a significant reduction in the percentages of infected cells, the numbers of tachyzoites and parasite-containing vacuoles per 100 cells at 24 h (Fig 7A). Knockdown of STAT3 resulted in a significant accumulation of LC3 and LAMP-1 around the parasite (Fig 7B and 7C). Moreover, silencing of Beclin 1 prevented induction of anti-*T*. *gondii* activity in cells subjected to STAT3 knockdown (Fig 7D). Next, we examined the role of phosphorylation of Y705 in parasite survival. Transfection of mouse endothelial cells with the non-phosphorylatable mutant Y705F STAT3 caused accumulation of LC3 around the parasite (Fig 7E). Expression of Y705F STAT3 in endothelial (Fig 7F) or epithelial cells (S4 Fig) resulted in a reduction in the percentage of infected cells and the numbers of tachyzoites per 100 cells at 24 h. Parasite killing was dependent on lysosomal enzymes since it was markedly inhibited by leupeptin/pepstatin (Fig 7F; S4 Fig). Thus, parasite-induced STAT3 signaling prevents *T*. *gondii* killing dependent on the autophagy machinery and lysosomal protease activity.

### *T*. *gondii*-induced FAK/Src/EGFR-induced STAT3 signaling prevents killing of *T*. *gondii* likely by preventing PKR and eIF2α activation

**Fig 8:**
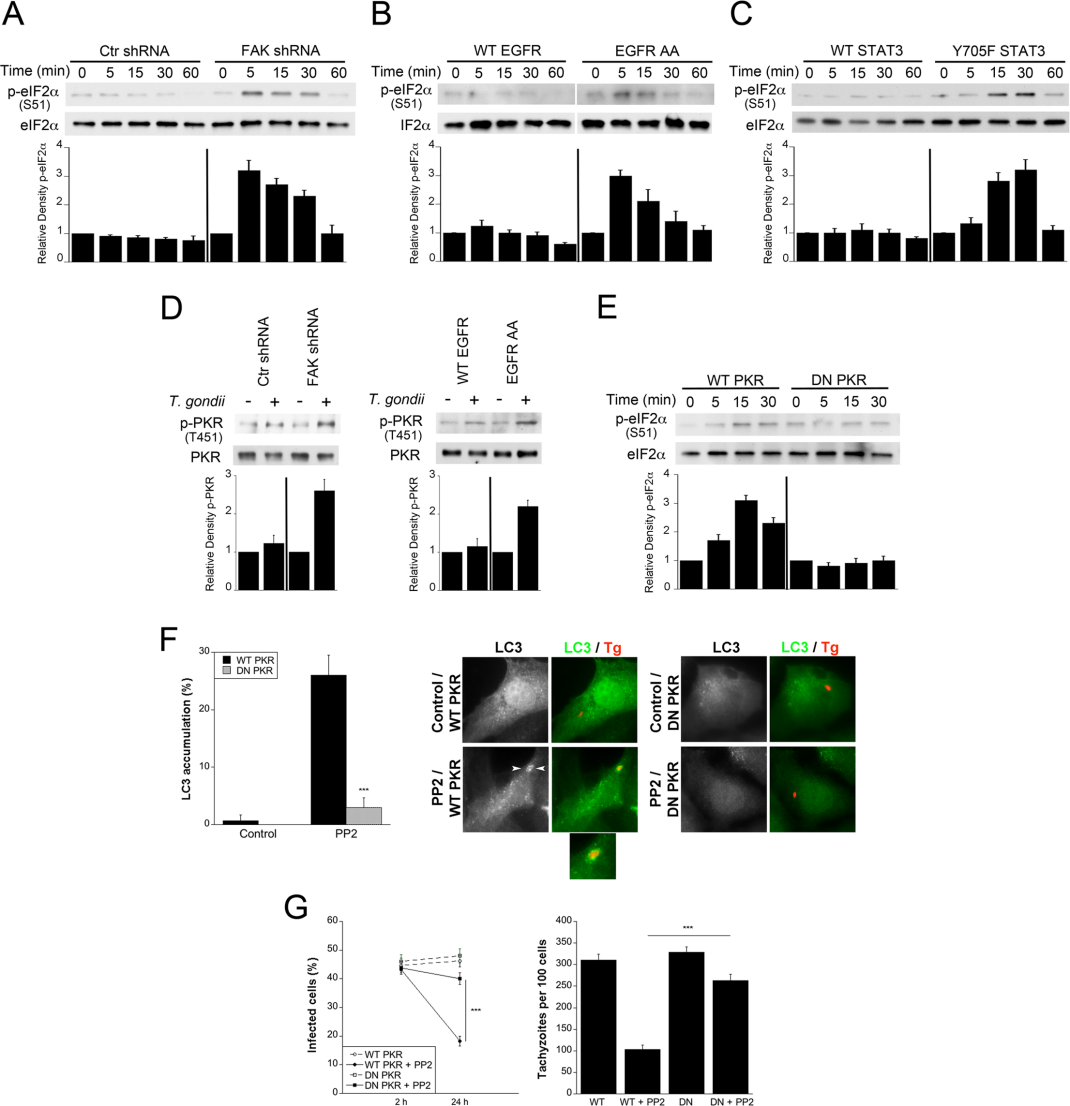
*T*. *gondii*-induced FAK/Src/EGFR-induced STAT3 signaling prevents autophagic killing of *T*. *gondii* likely by preventing PKR and eIF2α activation. *A-C*, mHEVc cells transduced with lentiviral vectors that express either FAK shRNA or control shRNA (*A*), NMuMG cells with stable expression of WT EGFR or EGFR AA mutant (*B*) or NMuMG cells with stable expression of WT EGFR cells transfected with WT STAT3 or Y705F STAT3 (*C*) were infected with RH *T*. *gondii*. Cell lysates were obtained to probe for total eIF2α and phospho-S51 eIF2α. Relative densities of phospho-eIF2α in lysates from infected cells were compared to those from their respective uninfected (control) cells. Relative density of phospho-eIF2α for uninfected samples was given a value of 1. *D*, mHEVc cells transduced with lentiviral vectors that express either FAK shRNA or control shRNA or NMuMG cells with stable expression of WT EGFR or EGFR AA mutant were challenged with RH *T*. *gondii*. Cell lysates were obtained at 5 min post-challenge to probe for total PKR and phospho-T451 PKR. Relative density of phospho-PKR was calculated as above. *E*, mHEVc cells transduced with lentiviral vectors that express FAK shRNA were transfected with WT or DN PKR followed by challenge with RH *T*. *gondii*. Cell lysates were probed for total eIF2α and phospho-S51 eIF2α. *F*, mHEVc cells were transfected with WT PKR or DN PKR followed by incubation with or without PP2 and infection with RFP-*T*. *gondii* (RH). Expression of LC3 was examined by immunofluorescence 5 h post-challenge. Arrowheads indicate accumulation of LC3 around the parasite. Original magnification X630. *G*, mHEVc cells transfected with WT PKR or DN PKR were treated with or without PP2, challenged with RH *T*. *gondii*. Monolayers were examined 2 and 24 h post-challenge. Results are shown as the mean ± SEM of 3 independent experiments. ** *P* < 0.01; *** *P* < 0.001.

S51 phosphorylation of eIF2α plays an important role in the stimulation of autophagy [41]. In addition, increased S51 phosphorylation of eIF2α accompanies autophagic killing of *T*. *gondii* induced by CD40 ligation [42]. Thus, we examined the effect FAK—Y845 EGFR—STAT3 signaling on activation of eIF2α. While infection with *T*. *gondii* did not significantly increase S51 phosphorylation of eIF2α in FAK-sufficient cells, knockdown of FAK increased eIF2α phosphorylation in cells infected with *T*. *gondii* (Fig 8A). Similarly, expression of the EGFR AA mutant (defective in Y845 phosphorylation) or transfection with Y705F-STAT3 resulted in increased S51 eIF2α phosphorylation in *T*. *gondii*-infected cells (Fig 8B and 8C). In addition, *T*. *gondii* induced marked eIF2α phosphorylation in EGFR-null CHO cells but not in EGFR^+^ CHO cells (S3B Fig). Next, we examined PKR signaling since this molecule is a major activator of eIF2α and autophagy, PKR links CD40 to autophagic killing of *T*. *gondii* and PKR is targeted by STAT3 to inhibit autophagy [40–43]. Knockdown of FAK or expression of EGFR AA increased T451 phosphorylation of PKR in cells infected with *T*. *gondii* (Fig 8D). Moreover, transfection with DN PKR impaired S51 eIF2α phosphorylation in *T*. *gondii*-infected cells that are deficient in FAK (Fig 8E). Finally, we examined whether killing of *T*. *gondii* required PKR signaling. The accumulation of LC3 around the parasite observed after inhibition of Src was ablated by transfection of DN PKR (Fig 8F). Moreover, expression of DN PKR also prevented parasite killing induced by inhibition of Src (Fig 8G). Taken together, these results suggest that *T*. *gondii* avoids autophagic killing by inducing cell signaling in host cells that prevents PKR and eIF2α activation.

## Discussion

**Fig 9:**
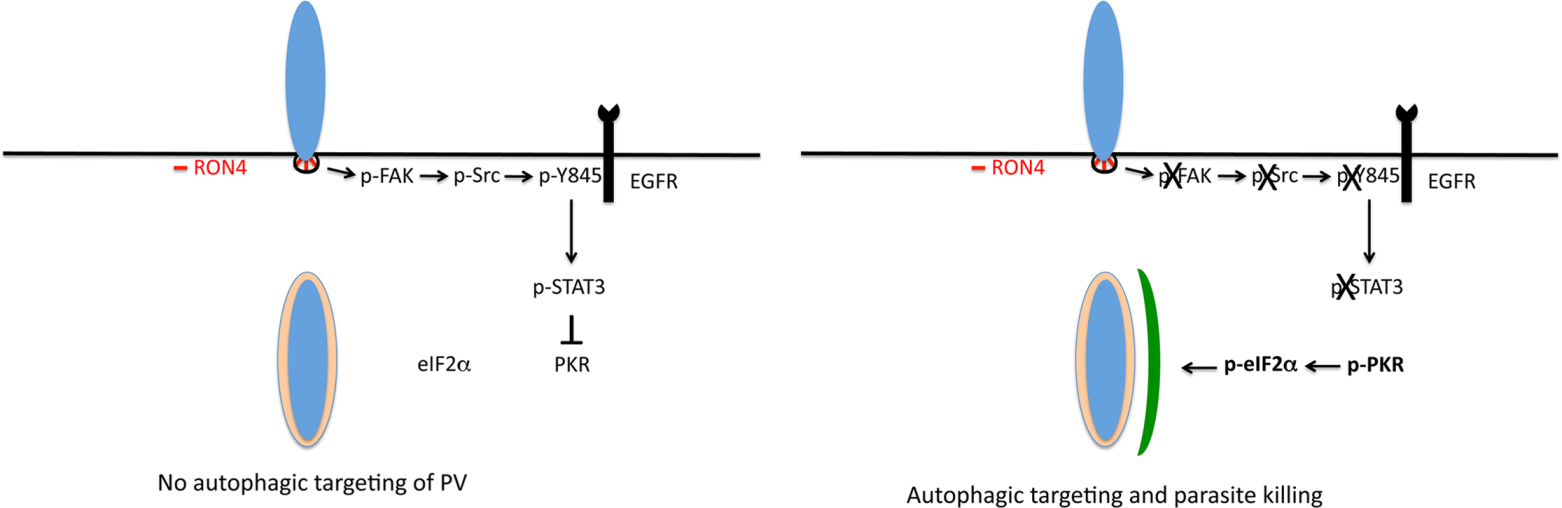
*T. gondii* invasion of host cells activates a signaling cascade that prevents autophagic targeting of the parasite. During invasion of mammalian cells, the formation of the moving junction, characterized by expression of RON4, appears to be accompanied by activation of FAK in the mammalian cell. In turn, FAK activates Src causing Src-dependent transactivation of EGFR (Y845 phosphorylation). This unique form of EGFR activation recruits STAT3 signaling that prevents activation of PKR and eIF2α. Blockade of the signaling cascade would result on activation of PKR and eIF2α leading to the formation of an autophagosome around the parasitophorous vacuole and killing of the parasite mediated by canonical autophagy.

Intracellular survival of *T*. *gondii* requires that the parasite avoids being targeted by autophagy, a process that would otherwise lead to lysosomal killing of the parasite. Herein we identified a pathway by which *T*. *gondii* prevents autophagic targeting (Fig 9). We report that during the process of invasion of mammalian cells, *T*. *gondii* induced expression of phospho Y397 FAK that appeared to associate in the host cell with RON4, a parasite protein expressed in the moving junction. In turn, FAK triggered Src activation and Src-mediated EGFR transactivation resulting in STAT3 signaling. FAK—Src—Y845 EGFR—STAT3 signaling induced by *T*. *gondii* was relevant because it prevented targeting of the parasite by the autophagy machinery. Inhibition of the components of this cascade in a broad range of resting host cells resulted in accumulation of LC3 and LAMP-1 around the PV and parasite killing that was dependent on ULK1, Beclin 1 and lysosomal protease activity. Moreover, inhibition of parasite-induced cell signaling enhanced phosphorylation of PKR and eIF2α. These events were relevant since the killing of *T*. *gondii* was ablated by expression of DN PKR. Taken together, these findings identified molecular events set in motion during the process of active invasion of host cells that enable *T*. *gondii* to restrict activation of PKR and eIF2α, molecules that are key drivers of autophagy.

FAK is a cytoplasmic molecule that responds to extracellular signals and regulates cell motility, survival and proliferation [44]. These effects are mediated by its function as a scaffold protein that interacts and phosphorylates other signaling molecules. Our studies indicate that invasion of mammalian cells by *T*. *gondii* caused activation of FAK and its interacting partner Src. EGFR was not required for activation of these signaling molecules. FAK can become activated by mechanical stimulation and integrin clustering [45]. The studies with the cRGDfV peptide, neutralizing anti-α_v_β3 Ab and knockdown of β1 integrin suggest that FAK signaling induced by *T*. *gondii* infection is driven to a large extent by β integrins, presumably in the form of mechano-transduction-induced integrin clustering at the site of host cell penetration causing activation of mammalian cytoplasmic FAK. While host cell invasion is driven by the *T*. *gondii* glideosome, the host cell also contributes to this process by forming an F-actin ring underneath the moving junction and accumulating microtubules at this site that would anchor the moving junction to the host cell cytoskeleton during invasion [46–48]. The studies presented herein revealed that the process of invasion not only induces rearrangement of host cell cytoskeleton but triggers FAK-Src signaling that plays a critical role in promoting parasite survival.

Src regulates various cellular processes through interactions with signaling proteins including EGFR. Indeed, binding of Src to EGFR can trigger EGFR signaling even in the absence of ligand binding [49]. While binding of EGF to EGFR induces autophosphorylation at tyrosine sites in the C-terminal non-catalytic domain of the receptor that act as docking sites for various signaling molecules, Src transactivation of EGFR is characterized by phosphorylation at a unique Y845 site located in the kinase domain of EGFR that leads to activation of alternate signaling molecules [31–34]. We reported that, through the effect of MIC3 and MIC6 (adhesins with EGF-like domains), *T*. *gondii* induces EGFR autophosphorylation and Akt activation that prevents autophagic targeting of the PV [9]. In contrast, Src-dependent Y845 phosphorylation of EGFR was critical for parasite-induced STAT3 activation but played no significant role in *T*. *gondii*-induced early Akt signaling. These results differ from those reported in cells exposed to HIV Tat or cells infected with *Salmonella typhimurium*, where both HIV Tat-induced Src activation and bacterial-induced FAK activation signal through Akt to inhibit autophagy [39, 50]. Our studies support the existence of 2 pathways by which *T*. *gondii* impairs autophagic targeting, FAK→Src→Y845-EGFR→STAT3 and MIC3/6→EGFR autophosphorylation→Akt.

Our studies indicate that *T*. *gondii* induces rapid STAT3 Y705 phosphorylation dependent on FAK—Src and Y845 EGFR phosphorylation. Knockdown of STAT3 and expression of Y705F STAT3 revealed that *T*. *gondii* activates STAT3 to prevent autophagic killing. While phosphorylated STAT3 is a well-recognized transcriptional regulator, STAT3 can also signal in the cytoplasm. Phosphorylated STAT3 crosstalks with signaling molecules in the cytoplasm and has been proposed to modulate signaling at this level [51–53]. We report that in *T*. *gondii*-infected cells, expression of Y705F STAT3 or inhibition of upstream inducers of STAT3 Y705 phosphorylation resulted in increased phosphorylation of PKR and/or eIF2α. Moreover, the studies using DN PKR indicate that targeting by LC3 and parasite killing triggered by inhibition of host cell signaling was dependent on PKR. STAT3 can inhibit autophagy through constitutive binding to PKR that prevents PKR activation, an effect that does not require STAT3 phosphorylation at Y705 [40]. Our studies suggest an additional mechanism by which STAT3 Y705 phosphorylation induced by *T*. *gondii* impairs PKR-eIF2α signaling. STAT3 is also reported to inhibit autophagy through increased transcription of negative regulators of autophagy [54]. Moreover, S727 phosphorylation of STAT3 promotes its mitochondrial localization and is reported to increase autophagy [55]. Altogether, STAT3 appears to regulate autophagy through various mechanisms, the nature of which may be determined in a context-dependent manner.

After being injected into the host cell during invasion, the *T*. *gondii* protein ROP16 migrates to the nucleus of the host cell and induces STAT3/6 activation that in turn impairs expression of IL-12, a cytokine that confers host protection against *T*. *gondii* [37, 38]. However, the effect of ROP16 on STAT3 phosphorylation is not noticeable until after 1–1.5 hr post-infection ([38] and this study) and the parasite induces STAT3 activation as early as 2 min post-infection [36]. The work herein identified FAK—Src—EGFR Y845 phosphorylation as a novel upstream inducer of STAT3 activation during *T*. *gondii* infection. It appears that this pathway rather than the ROP16 –STAT3 pathway is a major inhibitor of autophagic targeting of *T*. *gondii*. Taken together, *T*. *gondii* has evolved distinct mechanisms to activate STAT3 and through them sets in motion processes that impair the autophagic killing of the parasite and Th1 immunity.

Our studies identified a molecular cascade that appears to be associated with the formation of the moving junction during parasite invasion that leads to activation of STAT3 preventing targeting of the parasite by the autophagy machinery. Moreover, these studies suggest that parasite-induced STAT3 signaling functions by preventing activation of PKR and eIF2α. The demonstration that immune (CD40)-mediated induction of autophagic killing of *T*. *gondii* is dependent on activation of PKR [42] suggests that the intracellular survival of *T*. *gondii* may be affected by opposing effects of the parasite and cell mediated immunity on key components of autophagy signaling. Given that autophagy promotes *in vivo* protection against *T*. *gondii* [42, 56], pharmacologic approaches to prevent the parasite from activating host cell signaling that counter-regulates autophagy may result in novel treatment against toxoplasmosis.

## Materials and methods

### Mammalian cells

Human retinal pigment epithelial (RPE) cells (ARPE-19), human lung epithelial cells (A549) (both from American Type Culture Collection, ATCC, Manassas, VA), mouse high endothelial venule cells (mHEVc, gift from Joan Cook-Mills, Northwestern University), mouse microglia cell line (BV-2, gift from Kalipada Pahan, University of Nebraska), MDA-MB-231 human breast epithelial cells (ATCC) [30] and normal mouse mammary gland (NMuMG) epithelial cells (ATCC) [35] were cultured in either RPMI or DMEM plus 10% fetal bovine serum (FBS; HyClone, Logan, UT). NMuMG with stable expression of human WT EGFR or mutant EGFR where 679,680-LL was converted to AA (EGFR AA) were previously described [35]. Chinese Hamster Ovary (CHO; gift from Cathleen Carlin, Case Western Reserve University) cells were cultured in MEM plus 10% FBS.

### *T*. *gondii* and infection

Tachyzoites of the *T*. *gondii* strains RH (Type I strain), RH-YFP, RH-RFP, RH ROP16 knockout (*Δ*r*op16* [19]), RH ROP18 knockout (*Δ*r*op18*), their corresponding WT controls, (gifts from John Boothroyd, Stanford University, Stanford, CA), inducible conditional MIC8 knockout (MIC8KOi) (gift from Markus Meissner, University of Glasgow), ME49 (Type II strain) or VAND (atypical strain, BEI Resources, Manassas, VA) were maintained in human foreskin fibroblasts (ATCC). MIC8KOi parasites were cultured in HFF in the presence of anhydrotetracycline (1 μg/ml) for 48 h to deplete MIC8. *T*. *gondii* infection was synchronized using potassium buffer shift. Mammalian cells were incubated with the Src inhibitor PP2 (0.2 μM; Sigma-Aldrich, St. Louis, MO), FAK inhibitor PF-573228 (1 μM; Pfizer, Inc., New York, NY; both 1 h prior to challenge with *T*. *gondii*), integrin blocking peptide cRGDfV or control peptide cRADfV (3 μM; Bachem, Torrance, CA; 1 h prior to challenge), neutralizing anti-α_v_β3 mAb LM609 (15 μg/ml; EMD Millipore, Temecula, CA; 1 h prior to challenge), pertussis toxin (PTx; 100 ng/ml; EMD Millipore; 4 h prior to challenge), lysosomal inhibitors leupeptin and pepstatin (both 10 μM; EMD Millipore; 1 h after challenge with *T*. *gondii*). Parasite load was assessed as described [9]. In certain experiments cells were incubated with lysophosphatidic acid (LPA; 10 μM; Sigma Chemical, St. Louis, MO). None of the reagents described above affected cell viability (trypan blue exclusion).

### Transfections and lentiviral vectors

Cells were transfected with Src siRNA [57], ULK1 siRNA (Life Technologies), Beclin 1 siRNA [58], STAT3 siRNA [59], MyD88 siRNA [60] or control siRNA (Dharmacon, Lafayette, CO) as well as plasmids encoding WT EGFR, Y845F EGFR [32], WT STAT3, Y705 STAT3 (gift from Jim Darnell; Addgene plasmids # 8707 and 8709) [61], FLAG-tagged WT-PKR, dominant negative (DN)-PKR (K296R) or empty plasmid (gifts from Bill Sugden, University of Wisconsin) using Lipofectamine 2000 (Invitrogen, Carlsbad, CA) following manufacturer’s instructions. mHEVc cells were transduced with lentivirus vectors encoding FAK or control shRNA (Open Biosystems, Lafayette, CO) followed by selection with puromycin (10 μg/ml; Sigma-Aldrich). MDA-MB-231 cells transduced with lentiviral vectors encoding shRNA against β1 integrin or control shRNA were previously described [30].

### Fluorescence microscopy

Mammalian cells challenged with RFP *T*. *gondii* were fixed with 4% paraformaldehyde at 5 h and stained with rabbit anti-LC3 antibody (MBL International, Woburn, MA) plus Alexa 488-conjugated secondary antibody (Invitrogen, Carlsbad, CA). Mammalian cells infected with YFP *T*. *gondii* were fixed at 8 h and stained with mouse anti-human LAMP-1 mAb (Developmental Studies Hybridoma Bank; Iowa City, IA) plus Alexa 555-conjugated secondary antibody (Jackson ImmunoResearch Laboratories Inc.). Accumulation of LC3 or LAMP-1 around *T*. *gondii* was defined as the presence of a ring-like structure that surrounds the parasite [62, 63]. At least 50 cells per well (duplicate or triplicate wells per group per experiment) were counted manually. CHO cells were challenged with YFP *T*. *gondii* and fixed at 3 min. Monolayers were stained with mouse anti-phospho-Y397 FAK antibody (BD Biosciences) plus Alexa 555-conjugated secondary antibody (Jackson ImmunoResearch Laboratories Inc.) and rabbit anti-RON4 antibody (gift from John Boothroyd) followed by incubation with goat anti-rabbit Alexa 647-conjugated secondary antibody (Jackson ImmunoResearch Laboratories Inc.). Specificity of staining was determined by incubating monolayers with secondary antibody alone. Slides were analyzed using Leica DMI 6000 B automated microscope equipped for epifluorescence microscopy.

### Immunoblot

Membranes were probed with antibodies against Src, phospho-Y416 Src (all from Cell Signaling Technology, Danvers, MA), EGFR (Santa Cruz Biotechnology, Santa Cruz, CA), phospho-Y845 EGFR (Cell Signaling), FAK (Santa Cruz Biotechnology), phospho-Y397 FAK (BD Biosciences, San Jose, CA), STAT3 (Cell Signaling), phospho-Y705 STAT3 (Cell Signaling), eIF2α (Cell Signaling), phospho-S51 eIF2α (Cell Signaling), PKR (Santa Cruz Biotechnology), phospho-T451 PKR (Thermo Fisher Scientific, Waltham, MA), Beclin 1 (BD Biosciences), ULK1 (Sigma-Aldrich), MyD88 (Cell Signaling) or actin (Santa Cruz Biotechnology) followed by incubation with secondary antibodies (Santa Cruz Biotechnology). Intensities of phosphorylated and total proteins were calculated using ImageJ (NIH). Phospho-protein signal was normalized to total protein signal, before normalizing it relative to the control or 0 min time-point condition.

### Statistics

Results from pooled experiments were analyzed for statistical significance using 2-tailed Student’s *t* test and ANOVA. Differences were considered statistically significant when *P* < 0.05.

## Supporting information

S1 Fig *T*. *gondii* induces Src activation independently of EGFR, G protein coupled receptors, MyD88, ROP16 and ROP18. *A*, CHO cells (EGFR null) were challenged with tachyzoites of the RH strain of *T*. *gondii*. Cell lysates were obtained to probe for total Src and phospho-Src Y416. *B*, A549 cells were treated with or without pertussis toxin (PTx; 100 ng/ml) prior to addition of lysophosphatidic acid (LPA; 10 μM) or challenge with RH *T*. *gondii*. Cell lysates were obtained to probe for total Akt, phospho-Akt S473, total Src and phospho-Src Y416. Normalized densitometry data represent means ± SEM of 3–5 experiments. A vertical line was inserted between densitometry data in lysates from control and PTx-treated cells to indicate that relative densities of phospho-Src from infected cells treated with or without PTx were compared to bands from their respective uninfected (control) cells. Relative density of phospho-Src for uninfected samples was given a value of 1. *C*, A549 cells were transfected with MyD88 siRNA or control siRNA followed by challenge with RH *T*. *gondii*. Densitometry data in lysates from cells transfected with control or MyD88 siRNA that were infected with *T*. *gondii* were compared to bands from their respective uninfected (control) cells. Densitometries for control bands were given a value of 1. *D*, A549 cells were challenged with *Δrop16*, *Δrop18 T*. *gondii* or their respective WT controls. Immunoblots and densitometries were assessed as above. Densitometry data represent means ± SEM of 3 experiments.
(TIF)

S2 Fig Inhibition of β integrin, FAK and Src signaling induces killing of *T*. *gondii*. *A*, RPE cells, mHEVc, and BV-2 were incubated with vehicle or PP2 (0.2 μM) prior to challenge with RH *T*. *gondii*. Monolayers were examined by light microscopy at 2 h and 24 h to determine the percentage of infected cells and at 24 h to ascertain the number of tachyzoites and parasite-containing vacuoles. *B*, mHEVc cells transduced with lentiviral vectors that express either FAK shRNA or control shRNA were incubated with RH or the VAND strain of *T*. *gondii*. *C*, RPE were incubated with or without cRADfV or cRGDfV prior to challenge with RH *T*. *gondii*. *D*, MDA-MB-231 human breast epithelial cells transduced with vector encoding control shRNA or β1 integrin shRNA were incubated with or without a neutralizing anti-α_v_β3 mAb prior to challenge with RH *T*. *gondii*. The percentage of infected cells and numbers of tachyzoites per 100 cells were assessed as above. Results are shown as the mean ± SEM of 3 independent experiments. ** *P* < 0.01; *** *P* < 0.001.
(TIF)

S3 Fig EGFR promotes *T*. *gondii*-induced early STAT3 phosphorylation and prevents eIF2α phosphorylation. CHO cells (EGFR null) or CHO stably transfected to express EGFR (CHO EGFR) were infected with RH *T*. *gondii*. *A*, Cell lysates were obtained to probe for total STAT3 and phospho-Y705 STAT3. Relative densities of phospho-STAT3 in lysates from infected cells were compared to those from their respective uninfected (control) cells. Relative density of phospho-STAT3 for uninfected samples was given a value of 1. *B*, Expression of total eIF2α and phospho-S51 eIF2α were examined by immunoblot. Relative densities of phospho-eIF2α in lysates from infected cells were compared to those from their respective uninfected (control) cells. Relative density of phospho-eIF2α for uninfected samples was given a value of 1. Densitometry data represent means ± SEM of 3 independent experiments.
(TIF)

S4 Fig Expression of Y705F STAT3 induces killing of *T*. *gondii*. NMuMG cells transfected with WT STAT3 or Y705F STAT3 were challenged with RH *T*. *gondii* followed by addition of leupeptin/pepstatin (lysosomal inhibitors, LI). Monolayers were examined at 2 and 24 h post-challenge. Results are shown as the mean ± SEM of 3 independent experiments. *** *P* < 0.001.
(TIF)
