# Supplementary figures and images for: Toxoplasma gondii induces FAK-Src-STAT3 signaling during infection of host cells that prevents parasite targeting by autophagy
Source: PLoS Pathog. 2017 Oct 16;13(10):e1006671. doi: 10.1371/journal.ppat.1006671 (PMC5658194; doi:10.1371/journal.ppat.1006671)

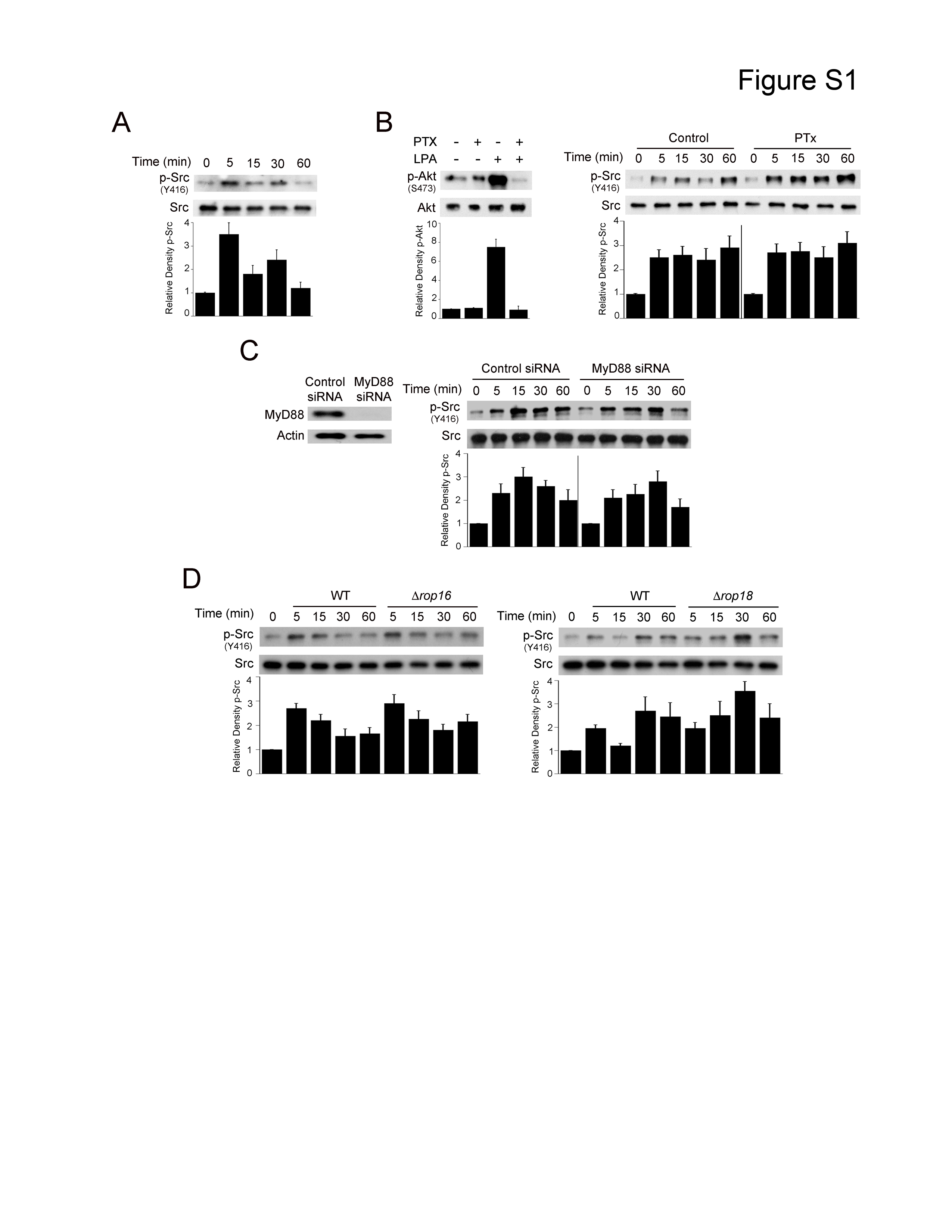

Supplement: S1 Fig — A, CHO cells (EGFR null) were challenged with tachyzoites of the RH strain of T. gondii. Cell lysates were obtained to probe for total Src and phospho-Src Y416. B, A549 cells were treated with or without pertussis toxin (PTx; 100 ng/ml) prior to addition of lysophosphatidic acid (LPA; 10 μM) or challenge with RH T. gondii. Cell lysates were obtained to probe for total Akt, phospho-Akt S473, total Src and phospho-Src Y416. Normalized densitometry data represent means ± SEM of 3–5 experiments. A vertical line was inserted between densitometry data in lysates from control and PTx-treated cells to indicate that relative densities of phospho-Src from infected cells treated with or without PTx were compared to bands from their respective uninfected (control) cells. Relative density of phospho-Src for uninfected samples was given a value of 1. C, A549 cells were transfected with MyD88 siRNA or control siRNA followed by challenge with RH T. gondii. Densitometry data in lysates from cells transfected with control or MyD88 siRNA that were infected with T. gondii were compared to bands from their respective uninfected (control) cells. Densitometries for control bands were given a value of 1. D, A549 cells were challenged with Δrop16, Δrop18 T. gondii or their respective WT controls. Immunoblots and densitometries were assessed as above. Densitometry data represent means ± SEM of 3 experiments. (TIF) [file ppat.1006671.s001.tif]

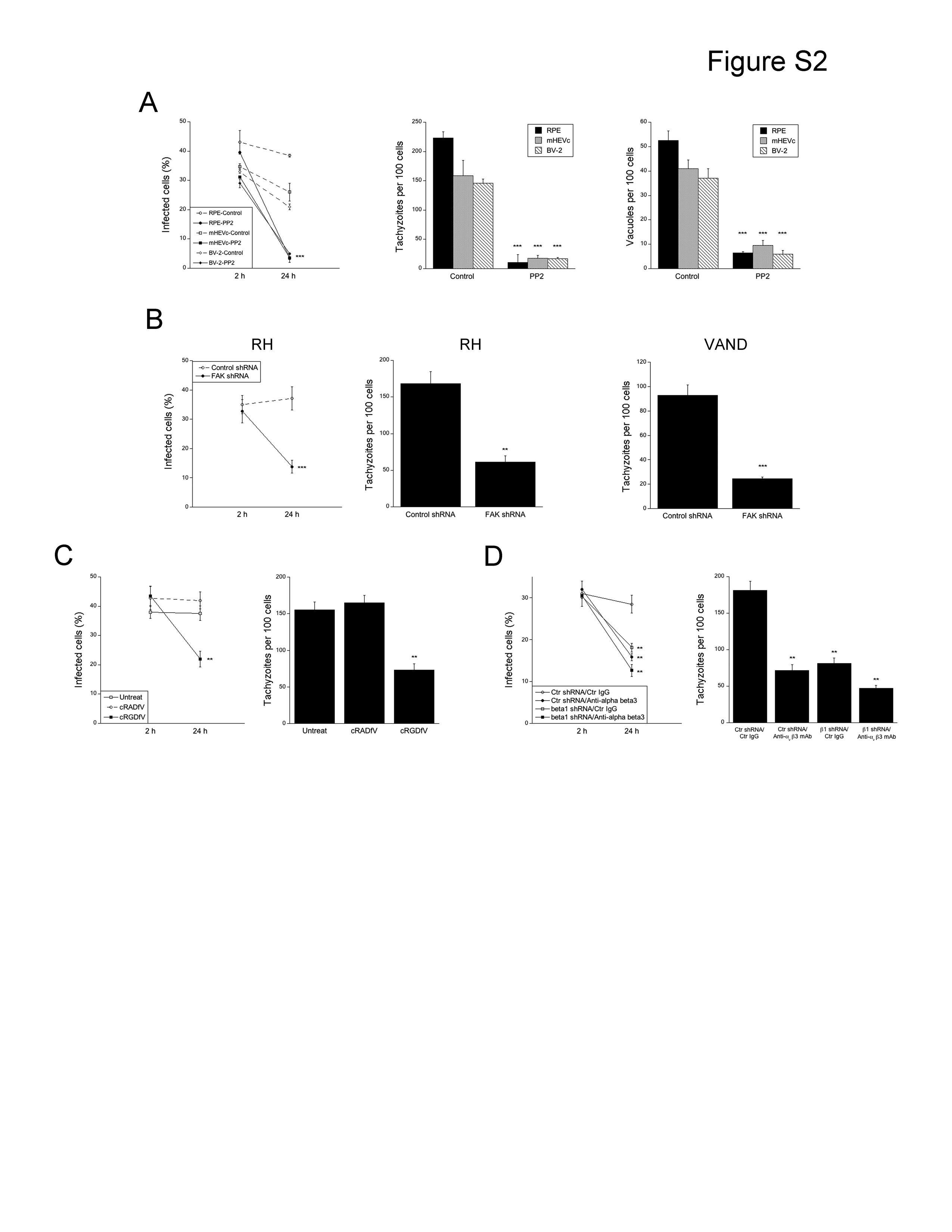

Supplement: S2 Fig — A, RPE cells, mHEVc, and BV-2 were incubated with vehicle or PP2 (0.2 μM) prior to challenge with RH T. gondii. Monolayers were examined by light microscopy at 2 h and 24 h to determine the percentage of infected cells and at 24 h to ascertain the number of tachyzoites and parasite-containing vacuoles. B, mHEVc cells transduced with lentiviral vectors that express either FAK shRNA or control shRNA were incubated with RH or the VAND strain of T. gondii. C, RPE were incubated with or without cRADfV or cRGDfV prior to challenge with RH T. gondii. D, MDA-MB-231 human breast epithelial cells transduced with vector encoding control shRNA or β1 integrin shRNA were incubated with or without a neutralizing anti-αvβ3 mAb prior to challenge with RH T. gondii. The percentage of infected cells and numbers of tachyzoites per 100 cells were assessed as above. Results are shown as the mean ± SEM of 3 independent experiments. ** P < 0.01; *** P < 0.001. (TIF) [file ppat.1006671.s002.tif]

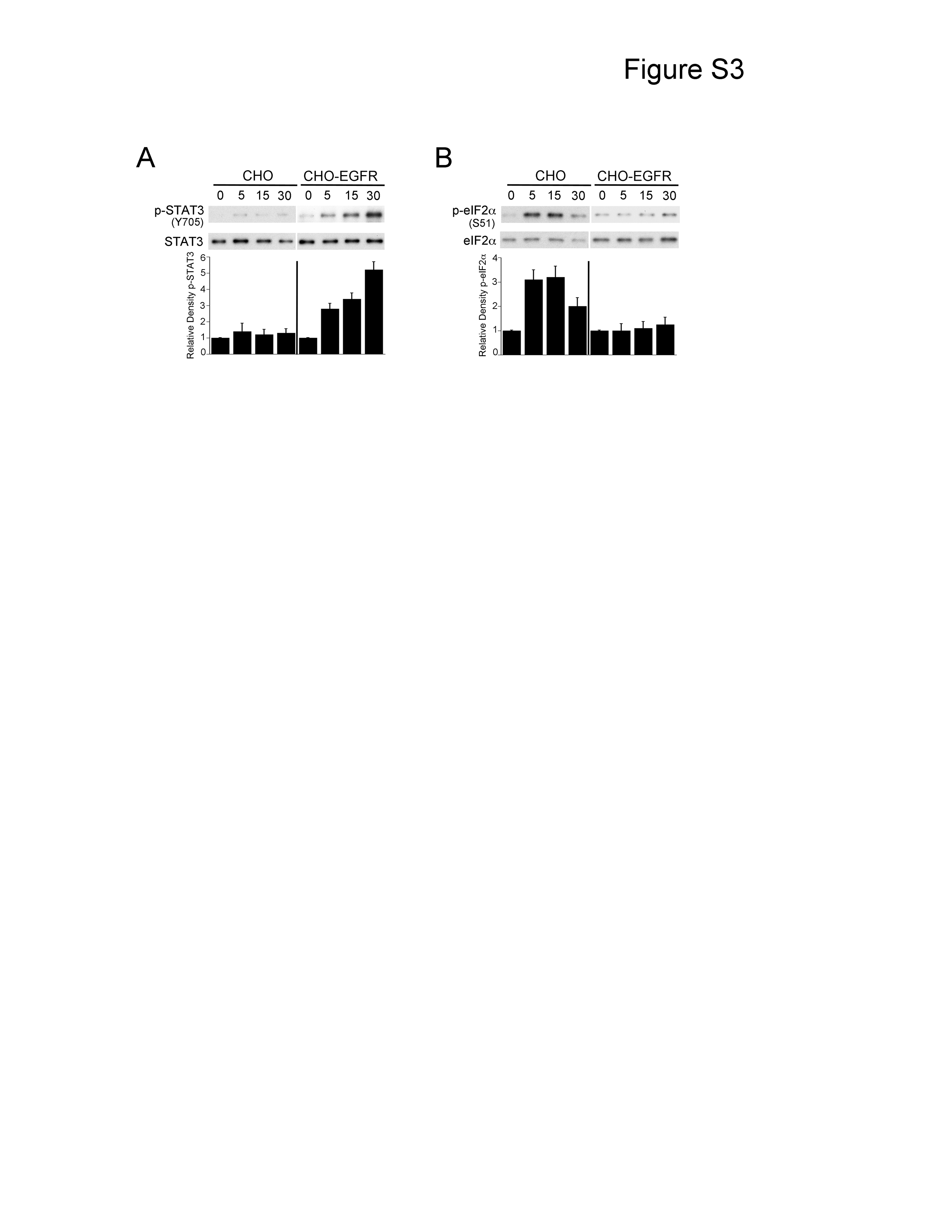

Supplement: S3 Fig — CHO cells (EGFR null) or CHO stably transfected to express EGFR (CHO EGFR) were infected with RH T. gondii. A, Cell lysates were obtained to probe for total STAT3 and phospho-Y705 STAT3. Relative densities of phospho-STAT3 in lysates from infected cells were compared to those from their respective uninfected (control) cells. Relative density of phospho-STAT3 for uninfected samples was given a value of 1. B, Expression of total eIF2α and phospho-S51 eIF2α were examined by immunoblot. Relative densities of phospho-eIF2α in lysates from infected cells were compared to those from their respective uninfected (control) cells. Relative density of phospho-eIF2α for uninfected samples was given a value of 1. Densitometry data represent means ± SEM of 3 independent experiments. (TIF) [file ppat.1006671.s003.tif]

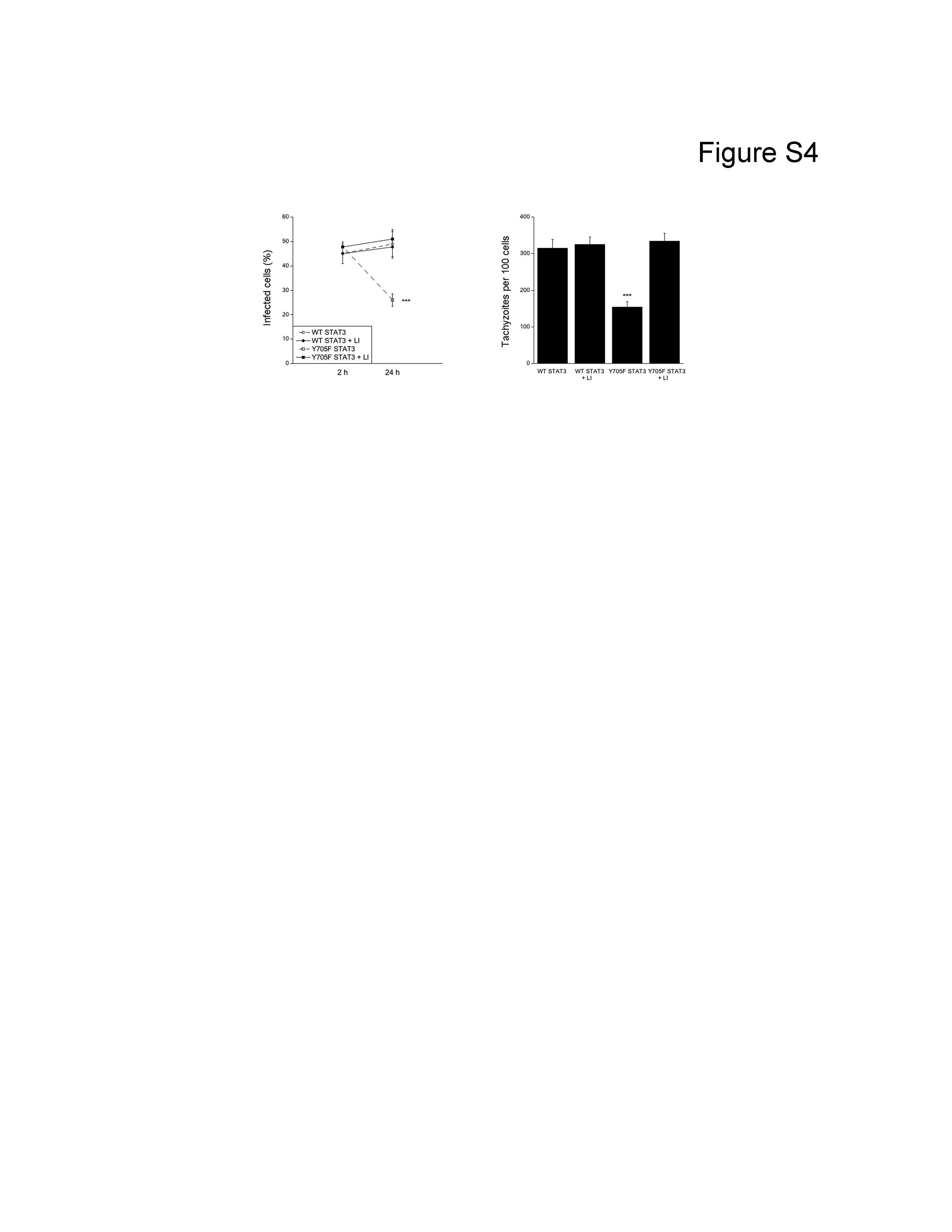

Supplement: S4 Fig — NMuMG cells transfected with WT STAT3 or Y705F STAT3 were challenged with RH T. gondii followed by addition of leupeptin/pepstatin (lysosomal inhibitors, LI). Monolayers were examined at 2 and 24 h post-challenge. Results are shown as the mean ± SEM of 3 independent experiments. *** P < 0.001. (TIF) [file ppat.1006671.s004.tif]
